# Consciousness doesn't overflow cognition

**DOI:** 10.3389/fpsyg.2014.01399

**Published:** 2014-12-04

**Authors:** Richard Brown

**Affiliations:** Philosophy Program, LaGuardia Community College, City University of New YorkLong Island City, NY, USA

**Keywords:** phenomenological overflow, higher-order thought theory, consciousness, partial report, fragile visual short-term memory

Theories of consciousness can be separated into those that see it as cognitive in nature, or as an aspect of cognitive functioning, and those that see consciousness as importantly distinct from any kind of cognitive functioning. One version of the former kind of theory is the higher-order-thought theory of consciousness. This family of theories posits a fundamental role for cognitive states, higher-order thought-like intentional states, in the explanation of conscious experience. These states are higher-order in that they represent the subject herself as being in various world-directed first-order states and thus constitute a kind of cognitive access to one's own mental life. This distinctive cognitive access is postulated to account for what it is like for one to have a conscious experience.

One important challenge to this approach is Block's case for phenomenological overflow (Block, 2007, 2011, 2012). The basic argument is that, overall, the balance of evidence favors the identification of phenomenal consciousness with first-order non-cognitive states rather than our cognitive access to those states. Emerging clearly from the ensuing debate is that Block's argument is meant to establish that phenomenology overflows *working memory*. This is important because, unlike other theories, the higher-order thought theory can allow that our conscious experience overflows working memory. In addition, it can account for the subjective impression that there is overflow even if there isn't.

Take the so-called Amsterdam paradigm (Sligte et al., 2008), which builds on Sperling's (1960) partial report paradigm. In these experiments, subjects are presented with a change-blindness-type scenario. For instance, they might be presented with a clock-like formation of rectangles. One array is presented followed by a variable interval and a second array, which may or may not contain a rectangle that had changed its orientation. Subjects are cued to the location of the potential change at various points during this process and then asked at the end if anything changed. Sligte et al. distinguish between what they call the “visual icon,” which is a highly detailed but brief positive afterimage occurring shortly after stimulus presentation, and what they call “fragile short-term memory,” which is less detailed but long-lasting. Subjects are able to perform the task successfully even when cued up to 6 s after the original presentation of the stimulus.

Block argues, largely on the basis on informal reports by subjects, that the best way to explain these findings is by positing a richly detailed phenomenally conscious experience of all of the shapes, rather than a sparsely detailed conscious experience corresponding to what is represented in working memory. Because the higher-order thought theory does not make the claim that encoding in working memory is required for conscious experience the theory could in principle accept this claim. The higher-order thought theory can allow that our phenomenal consciousness (that is, the contents of the relevant higher-order thoughts) overflows working memory. The relevant higher-order thoughts will be as detailed as the stream of consciousness, which, however sparse that is, will still be more detailed than what is encoded in working memory. What it cannot allow is that there is phenomenal consciousness in the absence of suitable higher-order thoughts instantiating a kind of cognitive access to the first-order states.

On the other end of the theoretical spectrum is the claim that only what is in working memory is phenomenally conscious and subjects are mistaken about the detail of their conscious experience. If so, then the conscious experience of subjects in the Amsterdam paradigm is to some degree generic, partial, fragmented, or degraded. The reports of “reading the answers off of conscious experience” may, to some extent, be confabulated. Subjects can do the task, they have the impression that they saw all of the rectangles, and they give a commonsense explanation. If this is the case then the higher-order theory will account for this by positing correspondingly fragmented, generic, or partial contents of the relevant higher-order states.

So at this point there may or may not be phenomenal consciousness that overflows working memory, but whatever the conscious experience of subjects in these experiments turns out to be we can explain it on the higher-order thought theory. This is because the higher-order thought theory makes the general claim that people may be aware of first-order states in virtue of some of the state's properties (that they are letters, that they are blocks, that they are arranged in various ways, that this particular block is oriented in that particular orientation, etc.), but not necessarily in virtue of all of their properties. Nonetheless, the information that the first-order states encode is causally efficacious. Higher-order-thought theories maintain that the *information* that is represented by the first-order state is partially unconscious, not that the first-order state itself is unconscious.

There is some evidence for this interpretation of the data from other work on change blindness. When subjects are not consciously aware of the difference between the two stimuli, both the original shape and the changed shape show priming effects; when the difference is consciously perceived only the changed stimulus shows those priming effects (Silverman and Mack, 2006). In both cases, subjects are aware of both stimuli, but being conscious of the difference between the two makes a difference in mental functioning. In addition, there is some evidence that subjects can detect such changes unconsciously (Fernandez-Duque and Thornton, 2000; Laloyaux et al., 2006).

Block interprets these claims as committing one to robust long-lasting unconscious working memory and he argues that the evidence currently doesn't support that hypothesis. For instance, he cites work by Soto et al. (2011) that suggests that unconscious working memory doesn't have the required capacity to explain the Amsterdam results. In this study, experimenters used masking to render a stimulus close to or below threshold and then asked subjects to compare a grating to a highly visible one presented up to 5 s later. Subjects were able to do it, but at a rate that is far below what subjects in the Amsterdam paradigm are capable of. This was the case even though the Soto task was much easier than that in the Amsterdam paradigm. In response to the criticism that the stimuli in the Soto experiments were masked, Block cites Carmel et al. (2011) which suggests that unconscious representations are short lived and so would not last the up to 6 s we find in the Amsterdam paradigm. Together these results suggest that unconscious working memory is not robust enough to explain the Amsterdam results.

Block raises a legitimate worry for those theories that do appeal to working memory, but it would be a mistake to lump the higher-order theory into that camp. As we have seen above, the higher-order-thought theory does not rely on unconscious states, but rather on some aspects of the targeted first-order states not being represented in the higher-order thought. We are aware of being in the states, and so they are conscious, but not in respect of all of their properties. By analogy compare what happens when I see a cardboard box, say, through a window but because the window is dirty I cannot make out what the box has written on it. I am aware of the box but not of all of its properties.

Another way to make the point is by stipulating a distinction between *phenomenal consciousness*—or there being something that there is like for the creature in question—and *state consciousness*—or being the target of a suitable higher-order representation (Brown, 2012, 2014). In the partial-report paradigm, the higher-order-thought theory claims that the first-order states, which are in fact the targets of the relevant higher-order representations, are state-conscious while the phenomenal consciousness of the subject is determined by the higher-order thought. In the Soto and Carmel et al. work, the relevant stimuli were all state-unconscious and so do not address the claim made by the higher-order thought theory. Subjects in the Amsterdam paradigm are maintaining a phenomenally conscious visual experience; most parties agree on that and even if one doesn't we can allow it for the sake of argument. What the higher-order theorist insists on is that this phenomenally conscious visual experience, which is determined by the content of the higher-order thought, may diverge from the informational content of the first-order states that are represented by the relevant higher-order states.

Block also appeals to work from Sligte et al. (2009), which found activity in V4 but not V1. This, he suggests, is not what we would expect if these representations were unconscious. However, it should now be clear that higher-order theory could allow that these states in V4 may be state-conscious. If these states are actually the first-order representations of the stimuli, then they are the targets of the higher-order cognitive access. The higher-order-thought theory claims that this cognitive access consists in thought-like states that result in one being aware of oneself as being in the relevant first-order states and since that cognitive access determines what it is like for you, what it is like for you will be relatively impoverished compared to “how it could have been,” so to speak, if more of the mental information carried by the first-order states was represented in the higher-order thought. For instance if one has a maximally determinate first-order representation of a grid and one's higher-order thoughts represent one as seeing only part of the grid then this is what it will be like for you. On the other hand if one is having a rich conscious experience as of the grid this will be because of the richness of the content of the relevant higher-order states. But in both cases the very same first-order states are state-conscious.

Thus, regardless of how the phenomenology of subjects turns out, the higher-order thought theory is well-situated to account for it. In fact, positing non-cognitive phenomenal consciousness itself comes with a high theoretical cost. Phenomenal consciousness consists in there being something that it is like *for* the subject of the experience and this suggests that there must be some kind of access to the experience, some kind of awareness of the experience as being one's own. Block has elsewhere argued that some non-cognitive form of awareness can account for this (Block, 2007), but no account of non-cognitive access to date can explain the subjective appearances.

Block does suggest a possible form of non-cognitive access. Following Sosa (2002) he offers a deflationary account on which we are aware of our mental states just in the having of them: just as we smile our own smiles just by smiling, so too we may experience our own experiences just by experiencing. When I feel a pain, not only do I experience the painful quality but I also experience it as mine. This is not the case when these states are unconscious. How can the deflationary account handle this? How is the mere having of one of these states different from the mere having of the other? While perhaps not an insurmountable problem, this is a formable obstacle to any non-cognitive account of awareness. Block also suggests the possibility of some kind of self-representational account. But there is no way to make sense of any such view except in higher-order terms. Block hasn't offered an alternative, but just appealed to there being one.

Some doubt that we can decide this issue in a theory neutral way (Kouider et al., 2012; Overgaard and Grunbaum, 2012) while others suggest that non-cognitive consciousness is somehow unscientific (Cohen and Dennett, 2011). I agree with Block (2012) that these views are mistaken. While it seems clear that we will never know with absolute certainty whether cognition plays a role in consciousness, we need not aspire to that unreachable goal. We should ask whether, within the confines of scientifically acceptable standards of evidence, the balance of available evidence favors one theory or another. I have been arguing that the higher-order thought theory is in a position to provide a more parsimonious “mesh” between psychology and neuroscience (Block, 2007; Lau and Brown, in press) but it also makes testable predictions.

If phenomenal consciousness depends in some way on higher-order cognitive functioning then we should be able to alter the conscious experience of subjects by interfering with areas of the brain thought to be involved in higher-order cognition while simultaneously leaving first-order processing unchanged or alternatively to produce conscious experience by directly stimulating the relevant areas (Weisberg, 2011). We might also expect that we could find cases where conscious experience outstrips first-order activity and that we would be able to “read-out” or “decode” this from activity in higher-order areas. In extreme conditions we would expect that we might find conscious experience in the absence of first-order sensory activity altogether. More work needs to be done but early attempts at testing these predictions have given suggestive results (Lau and Rosenthal, 2011; Lau and Brown, in press).

The higher-order thought theory of consciousness remains a reasonable working hypothesis with a slight edge against competing accounts and a robust research program to pursue.

## Conflict of interest statement

The author declares that the research was conducted in the absence of any commercial or financial relationships that could be construed as a potential conflict of interest.
